# Foliar Application of Chitosan (CTS), γ-Aminobutyric Acid (GABA), or Sodium Chloride (NaCl) Mitigates Summer Bentgrass Decline in the Subtropical Zone

**DOI:** 10.3390/plants13131773

**Published:** 2024-06-27

**Authors:** Bizhen Cheng, Qinyu Zhou, Linju Li, Muhammad Jawad Hassan, Weihang Zeng, Yan Peng, Zhou Li

**Affiliations:** Department of Turf Science and Engineering, College of Grassland Science and Technology, Sichuan Agricultural University, Chengdu 611130, China; chengbizhengrass@163.com (B.C.); 19982939311@163.com (Q.Z.); a15701939196@163.com (L.L.); jawadhassan3146@gmail.com (M.J.H.); zengwh0123@163.com (W.Z.)

**Keywords:** high temperature, photosynthesis, oxidative damage, osmotic adjustment, photochemical efficiency

## Abstract

Creeping bentgrass (*Agrostis stolonifera*) is an excellent cool-season turfgrass that is widely used in urban gardening, landscaping, and golf turf. Triennial field experiments from 2017 to 2019 were conducted to investigate effects of the foliar application of chitosan (CTS), γ-aminobutyric acid (GABA), or sodium chloride (NaCl) on mitigating summer bentgrass decline (SBD) and exploring the CTS, GABA, or NaCl regulatory mechanism of tolerance to summer heat stress associated with changes in chlorophyll (Chl) loss and photosynthetic capacity, osmotic adjustment (OA), oxidative damage, and cell membrane stability. The findings demonstrated that persistent ambient high temperatures above 30 °C during the summer months of 2017, 2018, and 2019 significantly reduced the turf quality (TQ), Chl content, photochemical efficiency of PSII (Fv/Fm and PI_ABS_), leaf relative water content, and osmotic potential (OP) but significantly increased electrolyte leakage (EL) and the accumulations of free proline, water-soluble carbohydrate (WSC), hydrogen peroxide (H_2_O_2_), and malondialdehyde (MDA). The foliar application of CTS, GABA, or NaCl could significantly alleviate SBD, as reflected by improved TQ and delayed Chl loss during hot summer months. Heat-induced declines in Fv/Fm, PI_ABS_, the net photosynthetic rate (Pn), the transpiration rate (Tr), and water use efficiency (WUE) could be significantly mitigated by the exogenous application of CTS, GABA, or NaCl. In addition, the foliar application of CTS, GABA, or NaCl also significantly improved the accumulations of free proline and WSC but reduced the EL, OP, and H_2_O_2_ content and the MDA content in leaves of creeping bentgrass in favor of water and redox homeostasis in summer. Based on the comprehensive evaluation of the subordinate function value analysis (SFVA), the CTS had the best effect on the mitigation of SBD, followed by GABA and NaCl in 2017, 2018, and 2019. The current study indicates that the foliar application of an appropriate dose of GABA, CTS, or NaCl provides a cost-effective strategy for mitigating SBD.

## 1. Introduction

With the development of increasing global warming worldwide, the negative impacts of high temperatures in hot summer on cool-season crop growth are increasing [1]. Creeping bentgrass (*Agrostis stolonifera*) is an excellent cold-season turfgrass with strong creeping growth characteristics, a delicate texture, and a low mowing tolerance. It is widely used in golf course putting green as one of the most popular turfgrasses all over the world [2,3,4]. However, high-temperature stress inhibits the growth of creeping bentgrass, accelerates leaf senescence, and reduces turf quality, ultimately leading to the improved cost of maintenance management [1,5,6]. Summer bentgrass decline (SBD) induced by hot summer has become an urgent problem that baffles golf course general managers in the golf industry [6]. Multiple measures have been applied to mitigate SBD such as the intelligent use of fertilizers, reasonable irrigation, and the foliar application of plant growth regulators (PGRs) [6,7,8,9]. Among them, the exogenous application of PGRs to alleviate SBD has many advantages, including their simple operation, low cost, fast effectiveness, and easy promotion and utilization.

γ-Aminobutyric acid (GABA) is a natural non-protein amino acid that is present in almost all living organisms [10]. Many studies have shown the beneficial roles of the exogenous application of an appropriate dose of GABA in improving heat tolerance in different plant species, including wheat (*Triticum aestivum*) seedlings, tea (*Camellia sinensis*) plants, mungbean (*Vigna radiata*) plants, and sunflower (*Helianthus annuus*) plants [11,12,13,14]. Our previous studies also found that the foliar application or supply of GABA in root zones could significantly mitigate heat-induced damage to creeping bentgrass by regulating antioxidant defense, metabolic homeostasis, and heat shock factor pathways [15,16,17]. In addition, as a low-priced and innoxious PGR, chitosan (CTS) has been widely used in agriculture and horticulture for improvements in crop and fruit yield and quality [4,18]. In cool-season grass species, exogenous CTS effectively improved the drought tolerance of annual ryegrass (*Lolium multiflorum*), white clover (*Trifolium repens*), and creeping bentgrass associated with enhanced antioxidant defense to reduce oxidative damage and carbohydrates metabolism for reduced osmotic potential (OP) [19,20,21]. A suitable dose of CTS could also significantly alleviate salt stress by regulating metabolic homeostasis and the transport of Na^+^ in the leaves and roots of creeping bentgrass [22]. In response to heat stress, creeping bentgrass pretreated with CTS maintained better growth and lower leaf senescence related to CTS-induced improvements in antioxidant capacity, chlorophyll (Chl) biosynthesis, and heat shock pathways [23,24]. These previous studies indicated a positive role of CTS in enhancing the heat tolerance of creeping bentgrass. However, the effects of the foliar application of GABA or CTS on mitigating SBD have not been investigated during hot summer yet.

Redundant sodium chloride (NaCl) is known as a critical soil pollutant that causes irreversible damage to plant growth and development [25]. The remediation of halomorphic soil and the rational utilization of salty soil for crop production are currently research hotspots [26]. On the other hand, it has been reported that Na^+^ acted as an important osmolyte in plant vacuoles and biostimulant to induce stress tolerance. For example, soybean (*Glycine max*) seedlings were exposed to a low concentration of a 34 mM NaCl solution in advance, which could effectively alleviate subsequent salt stress induced by a high concentration of 137 mM NaCl [27]. Seed priming with a low concentration of NaCl could significantly promote germination under drought stress because NaCl priming enhanced amylolysis and antioxidant defense to alleviate drought-induced oxidative damage [28]. Exogenous application of a low dose of NaCl could also ameliorate drought tolerance of white clover by improving Chl biosynthesis and reducing OP [29]. The study by Wijewardene et al. also demonstrated that transgenic *Arabidopsis thaliana* overexpressing an *AVP1* gene involved in the compartmentalization of Na^+^ into vacuoles exhibited significantly higher heat tolerance and tolerance to combined drought and heat stress compared to the wild type [30]. However, the beneficial effects of the foliar application of an appreciated concentration of NaCl on the heat tolerance of cool-season turfgrass and the alleviation of SBD during the hot summer months have not been reported.

The objectives of this study were to evaluate the effectiveness of three exogenous PGRs (GABA, CTS, or NaCl) in improving the turf quality (TQ) of creeping bentgrass during triennial hot summers and to further explore the physiological effects of the foliar application of GABA, CTS, and NaCl on mitigating SBD associated with changes in the photochemical efficiency of photosystem II, OP, and oxidative damage in a subtropical zone.

## 2. Results

### 2.1. Turf Quality Affected by the Foliar Application of GABA, NaCl, or CTS during Hot Summer

**Figure 1:**
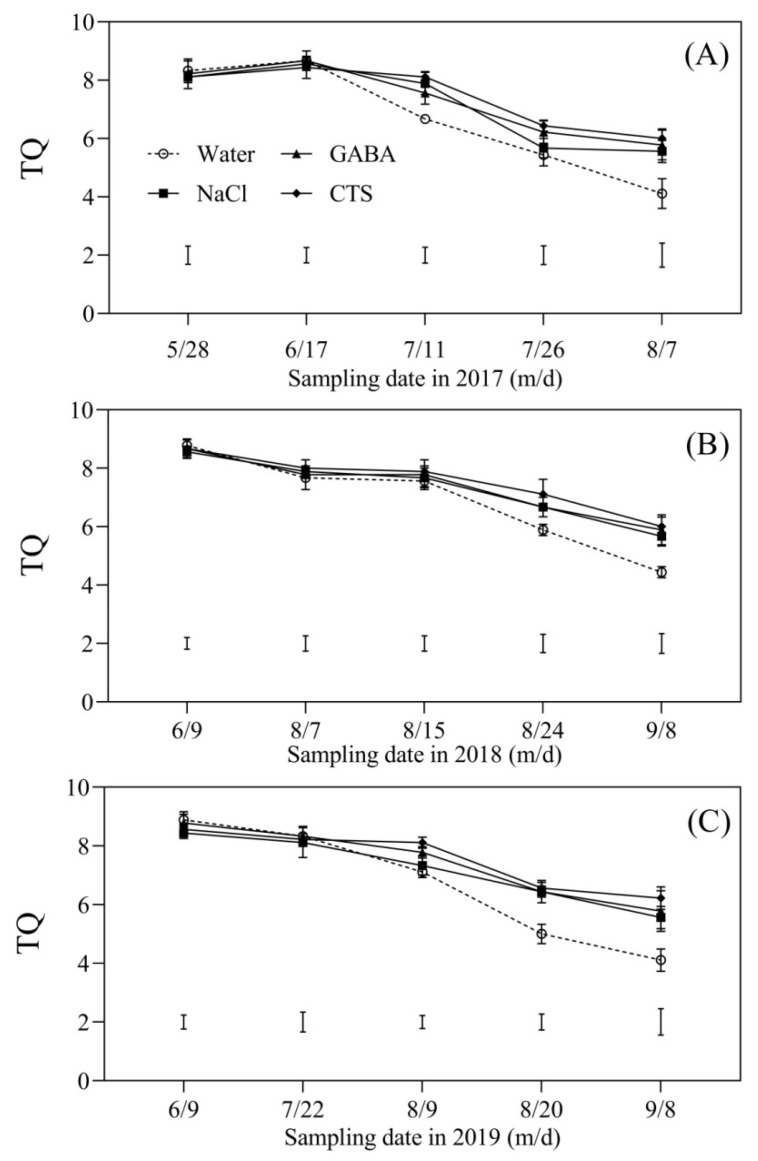
Effect of the foliar application of GABA, NaCl, CTS, or water on turf quality (TQ) during summer months in (**A**) 2017, (**B**) 2018, and (**C**) 2019.

During the summer months in 2017, 2018, and 2019, the TQ of four treatments gradually decreased (Figure 1A–C). On 7 August 2017, turfs sprayed with NaCl, GABA, or CTS exhibited significantly higher TQ than the turfs sprayed with water (Figure 1A). From 24 August to 8 September 2018 and 20 August to 8 September 2019, turfs sprayed with NaCl, GABA, or CTS also exhibited significantly higher TQ than non-PGR-treated turfs (Figure 1B,C).

### 2.2. Chlorophyll Content and Photosynthesis Affected by GABA, NaCl, or CTS during Hot Summer

**Figure 2:**
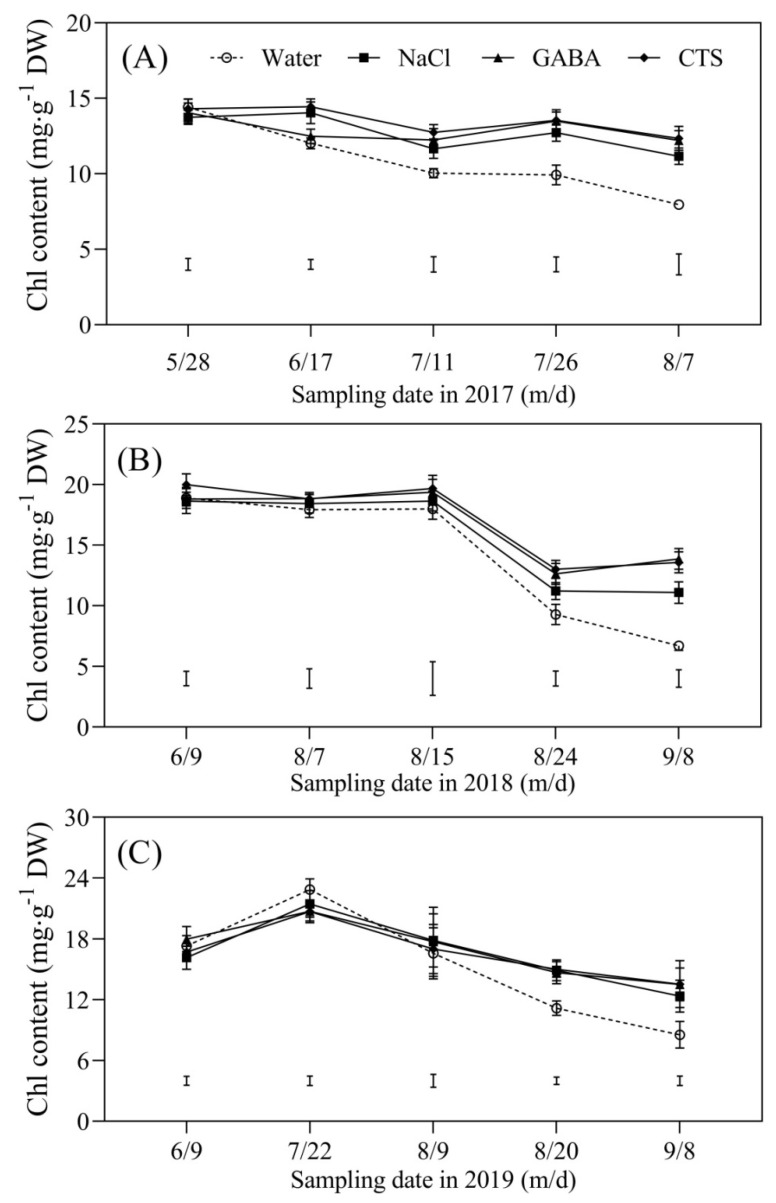
Effect of the foliar application of GABA, NaCl, CTS, or water on chlorophyll (Chl) content during summer months in (**A**) 2017, (**B**) 2018, and (**C**) 2019. Vertical bars indicate the least significant difference (LSD) values (*p* ≤ 0.05) on a given day.

**Figure 3:**
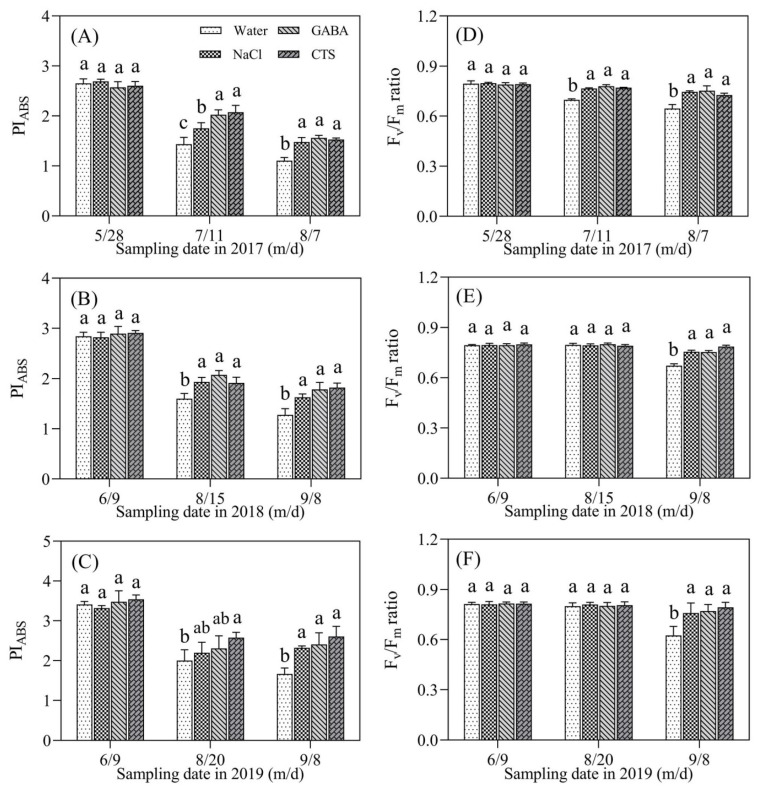
Effect of the foliar application of GABA, NaCl, CTS, or water on (**A**–**C**) photochemical efficiency (Fv/Fm) and (**D**–**F**) the performance index on an absorption basis (PI_ABS_) during summer months in 2017, 2018, and 2019. Vertical bars above the columns indicate ± SD of the mean (n = 3). Different letters indicate significant differences (*p* ≤ 0.05) on a given day.

Chl content in NaCl-, GABA-, or CTS-treated turfs was maintained at significantly higher levels from 28 May to 7 August 2017 but gradually declined in water-treated turfs during this time (Figure 2A). There was no significant difference in Chl content among the four treatments from 9 June to 15 August 2018. Turfs sprayed with exogenous NaCl, GABA, or CTS had significantly higher Chl content than turfs sprayed with water from 24 August to 8 September 2018 (Figure 2B). Chl contents of the four treatments significantly declined from 22 July to 8 September 2019, whereas the exogenous application of NaCl, GABA, or CTS significantly improved Chl content from 20 August to 8 September 2019 (Figure 2C). Summer heat stress induced a significant decline in PI_ABS_ in all treatments in 2017, 2018, and 2019 (Figure 3A–C). The exogenous application of GABA, NaCl, or CTS significantly increased the PI_ABS_ in 2017 (11 July and 7 August), 2018 (15 August and 8 September), and 2019 (8 September) (Figure 3A–C). On 20 August 2019, the foliar application of the CTS treatments significantly improved PI_ABS_, but there was no significant difference in PI_ABS_ among NaCl-, GABA-, and water-treated turfs (Figure 3C). The GABA, NaCl, or CTS treatment had also significantly higher Fv/Fm than water treatment in the summers of 2017 (11 July and 7 August), 2018 (8 September), and 2019 (8 September) (Figure 3D–F).

**Figure 4:**
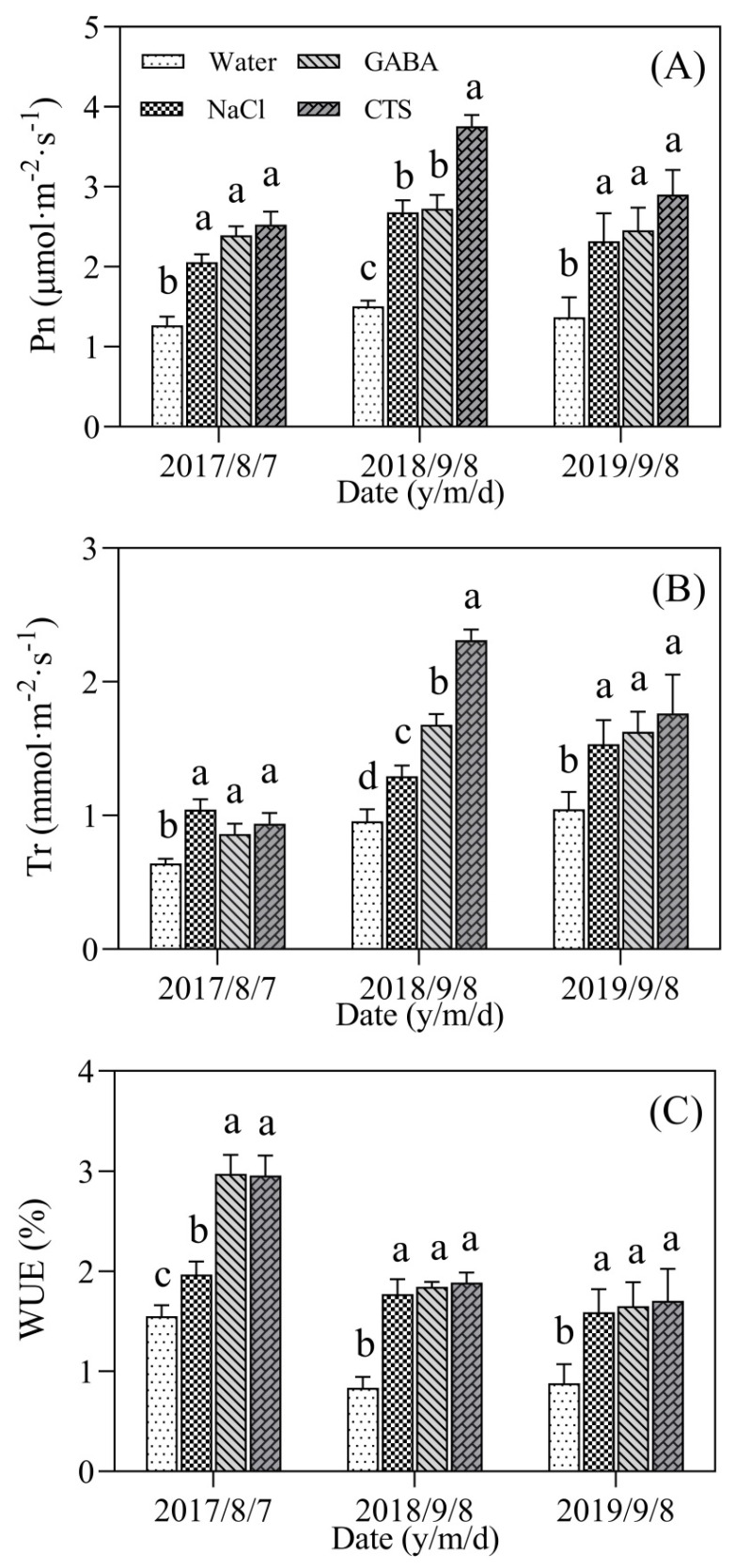
Effect of the foliar application of GABA, NaCl, CTS, or water on (**A**) the net photosynthetic rate (Pn), (**B**) transpiration rate (Tr), and (**C**) water use efficiency (WUE) during summer months in 2017, 2018, and 2019. Vertical bars above columns indicate ± SD of the mean (n = 3). Different letters indicate significant differences (*p* ≤ 0.05) on a given day.

On 7 August 2017, the NaCl, GABA, and CTS treatment had a 63%, 89%, and 99% significant increase in Pn compared to the water treatment, respectively (Figure 4A). On 8 September 2018, the CTS treatment exhibited the highest Pn compared to the other treatments, and the NaCl and GABA treatments also had a 78% and 81% significant increase in Pn compared to the water treatment in summer (Figure 4A). On 8 September 2019, the NaCl-, GABA-, or CTS-treated turfs also maintained significantly higher Pn than the untreated turfs (Figure 4A). PGR-treated turfs exhibited a 15% significant increase in Tr compared to the untreated turfs on 7 August 2017 (Figure 4B). In addition, the CTS-, GABA-, or NaCl-treated turfs had the highest, the second highest, or the third highest Tr on 8 September 2018 (Figure 4B). The foliar application of three PGRs also significantly improved Tr on 8 September 2019 (Figure 4B). The WUE of the turfs could be significantly improved by the exogenous application of NaCl, GABA, or CTS on 7 August 2017, 8 September 2018, and 8 September 2019 (Figure 4C).

### 2.3. Water Status and Osmolytes Affected by the Foliar Application of GABA, NaCl, or CTS during Hot Summer

**Figure 5:**
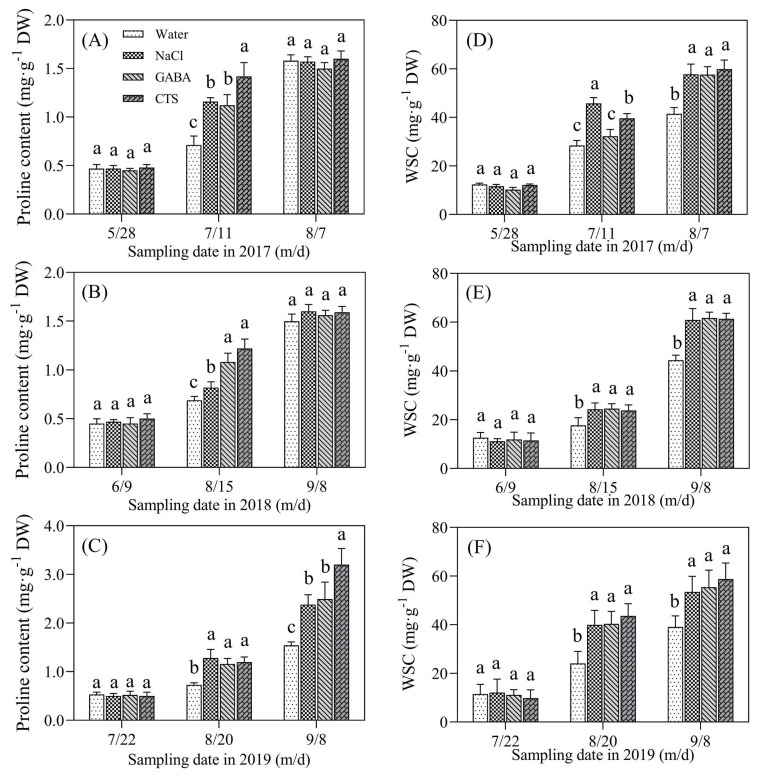
Effect of the foliar application of GABA, NaCl, CTS, or water on (**A**–**C**) proline (Pro) content and (**D**–**F**) water-soluble carbohydrate (WSC) during the summer months in 2017, 2018, and 2019. Vertical bars above the columns indicate ± SD of the mean (n = 3). Different letters indicate significant differences (*p* ≤ 0.05) on a given day.

**Figure 6:**
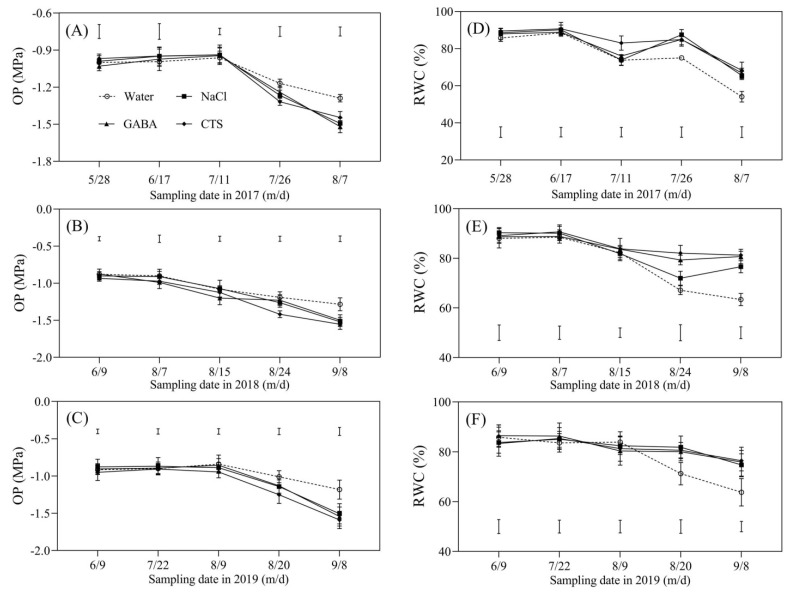
Effect of the foliar application of GABA, NaCl, CTS, or water on (**A**–**C**) osmotic potential (OP) and (**D**–**F**) relative water content (RWC) during the summer months in 2017, 2018, and 2019. Vertical bars indicate the least significant difference (LSD) values (*p* ≤ 0.05) on a given day.

Free proline content in all treatments gradually increased with the development of heat stress in the summers of 2017, 2018, and 2019 (Figure 5A–C). There was no significant difference in proline content among the four treatments on 28 May 2017, 9 June 2018, and 9 June 2019 (Figure 5A–C). On 11 July 2017 and 15 August 2018, the NaCl, GABA, and CTS treatments had significantly higher proline content than the water treatment, and the CTS treatment exhibited the highest proline content compared to the other treatments (Figure 5A,B). On 20 August and 8 September 2019, the foliar application of NaCl, GABA, and CTS significantly improved the accumulation of proline, and the CTS treatment exhibited the highest proline content compared to the other treatments on 8 September 2019 (Figure 5C). On 11 July 2017, the exogenous application of NaCl or CTS significantly increased the accumulation of WSC, but there were no significant differences in WSC content between the GABA treatment and the water treatment at this time (Figure 5D). The foliar application of NaCl, GABA, or CTS also could significantly increase the accumulation of WSC in leaves in 2017 (7 August), 2018 (15 August and 8 September), and 2019 (20 August and 8 September) (Figure 5D–F). Changes in RWC and OP in the leaves showed similar reduced trends among all treatments during the summer months of 2017, 2018, and 2019 (Figure 6A–C). The NaCl, GABA, and CTS treatments maintained a significantly lower OP and a higher RWC than the water treatment at the end of summer stress in 2017 (7 August), 2018 (8 September), and 2019 (8 September) (Figure 6A–F).

### 2.4. Oxidative Damage and Cell Membrane Stability Affected by the Foliar Application of GABA, NaCl, or CTS during Hot Summer

**Figure 7:**
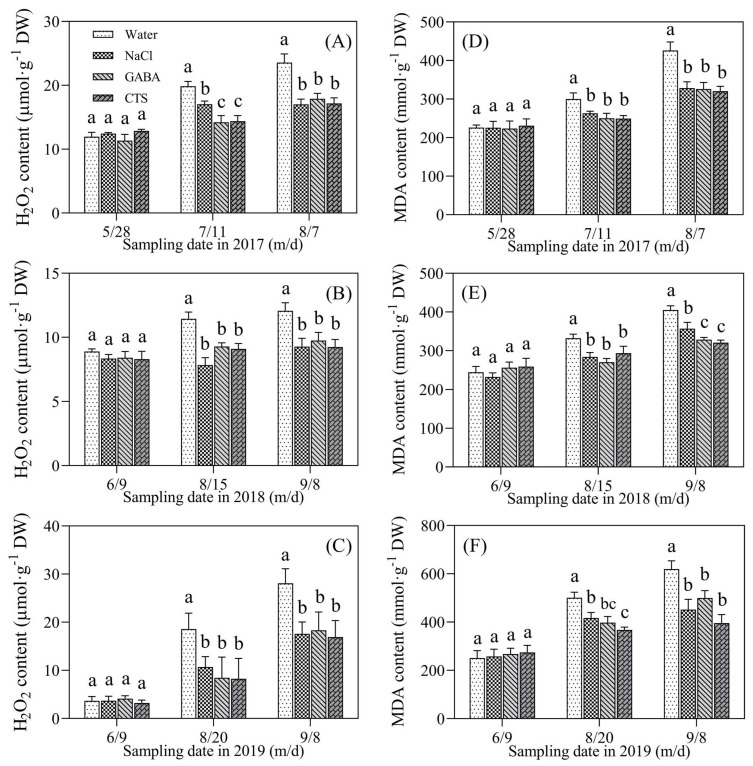
Effect of the foliar application of GABA, NaCl, CTS, or water on (**A**–**C**) hydrogen peroxide (H_2_O_2_) content and (**D**–**F**) malondialdehyde (MDA) content during the summer months in 2017, 2018, and 2019. Vertical bars above the columns indicate ± SD of the mean (n = 3). Different letters indicate significant differences (*p* ≤ 0.05) on a given day.

**Figure 8:**
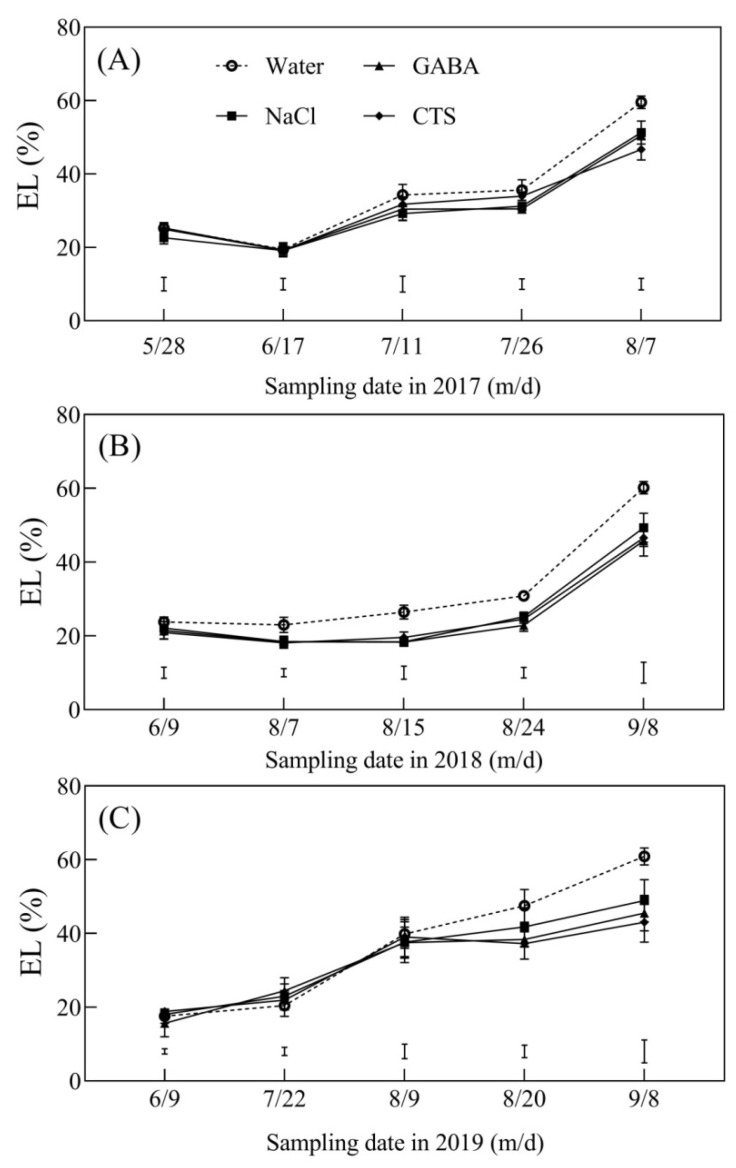
Effect of the foliar application of GABA, NaCl, CTS, or water on electrolyte leakage (EL) during the summer months in (**A**) 2017, (**B**) 2018, and (**C**) 2019. Vertical bars indicate the least significant difference (LSD) values (*p* ≤ 0.05) on a given day.

Accumulations of H_2_O_2_ and MDA in the leaves of all treatments showed no significant differences on 28 May 2017, 9 June 2018, and 9 June 2019 (Figure 7A–F). Summer heat stress induced significant increases in H_2_O_2_ and MDA contents in the four treatments (Figure 7A–F). The H_2_O_2_ and MDA contents in the GABA, NaCl, or CTS treatment were significantly lower than the water treatment in the summers of 2017 (11 July and 7 August), 2018 (15 August and 8 September), and 2019 (20 August and 8 September) (Figure 7A–C). The PGR-treated turfs had a 12% or a 23% significant decline in MDA content compared to the water treatment on 11 July or 7 August 2017, respectively (Figure 7D). Significantly lower MDA content was also detected in PGR-treated turfs compared to untreated turfs during the summer stress of 2018 and 2019 (Figure 7E,F). During the summer months of 2017, 2018, and 2019, the EL of four treatments gradually increased (Figure 8A–C). The foliar application of NaCl, GABA, or CTS significantly slowed down the increase in the EL induced by the summer heat stress in 2017, 2018, and 2019 (Figure 8A–C). The SFVA showed that the foliar application of CTS, GABA, or NaCl had the best, second-best, or third-best effect on alleviating SBD during the summer months of 2017, 2018, and 2019 (Table 1).

## 3. Discussion

During summer months, high temperature is one of the critical factors that significantly reduce the TQ of cold-season turfgrass worldwide [31]. As an excellent turfgrass for golf course putting greens, a high temperature above 30 °C not only decreases the TQ of creeping bentgrass but also increases maintenance inputs and costs against summer heat stress [6,32]. The application of exogenous PGRs is a simple and cost-effective approach to mitigating SBD. For example, the foliar application of trinexapac-ethyl (TE) and diethyl aminoethyl hexanoate (DA-6) could significantly improve the TQ of creeping bentgrass during hot summer months [9,33]. Although many previous studies have found that the exogenous application of GABA or CTS could significantly improve the heat tolerance of creeping bentgrass, beneficial effects of the foliar application of GABA or CTS on alleviating SBD have not been reported [4,34]. In addition, the exogenous application of appropriate doses of NaCl alleviated drought-induced damage to cool-season grass species, but the impact of NaCl on heat tolerance in plants and SBD is still not elucidated [28,29]. Our current findings demonstrated that the TQ of the four treatments (water, GABA, CTS, and NaCl) gradually declined during the summer months in 2017, 2018, and 2019. However, the declines in TQ induced by summer heat stress could be significantly mitigated by the foliar application of GABA, CTS, or NaCl, which indicated that the exogenous application of optimal doses of these PGRs could be an effective way to alleviate SBD in the subtropical zone. Based on the comprehensive evaluation of the SFVA, the CTS had the best effect on the mitigation of SBD, followed by GABA and NaCl in 2017, 2018, and 2019.

Classical symptoms of heat-reduced TQ are yellow and brown leaves, as well as low density and coverage [35]. A prolonged period of summer stress accelerates Chl loss, leading to reduced photosynthetic capacity, as reflected by decreases in Fv/Fm, PI_ABS_, and Pn in the leaves of creeping bentgrass [1,33]. It has been shown that the heat-induced decline in TQ was positively related to Chl loss [1,15,36]. During the summer months in 2017, 2018, and 2019, gradual decreases in the Chl content, Fv/Fm, PI_ABS_, and Pn in the leaves of creeping bentgrass were observed, which was consistent with the significant decrease in TQ. However, the foliar application of GABA, CTS, or NaCl significantly alleviated these negative effects of high-temperature stress, such as SBD, Chl loss, and the reduced photosynthetic capacity of creeping bentgrass, during summer months. In view of a cost-effective way, the foliar application of GABA, CTS, or NaCl provides an available strategy for mitigating SBD. The study by Rossi also found that the foliar application of GABA could effectively suppress heat-induced leaf senescence by reducing Chl degradation contributing to an improved TQ of creeping bentgrass in controlled-environment growth chambers [37], which supports our current findings.

The maintenance of stomatal opening is propitious to achieve heat dissipation through Tr, but the imbalance between Tr and root water absorption from soils leads to physiological drought due to increased leaf water loss and reduced root vitality under heat stress [1]. It has been reported that delayed declines in Tr and WUE could contribute to better heat tolerance in cool-season grass species [15,38,39]. A previous study found that heat damage to creeping bentgrass could be significantly alleviated by the exogenous application of GABA, which was associated with improved Tr and WUE for water homeostasis under controlled conditions [40]. In our current study, the foliar application of CTS, GABA, and NaCl significantly improved the RWC, Tr, and WUE of turfs during hot summer months, which indicated that the GABA-, CTS-, or NaCl-enhanced thermotolerance of creeping bentgrass turfs was associated with better heat dissipation and water homeostasis in summer.

In addition, water balance also depends on the change in osmotic adjustment (OA) when plants suffer from water-deficit stresses, such as drought and lenghty periods of high temperatures [21,41]. The accumulation of WSC not only enhances the energy supply for plant growth and development but also acts as an important osmolyte for OA and scavenging of free radicals under stressful conditions [21,42,43]. Proline is best known as a stress-induced osmoregulation substance, exhibiting dual functions of OA and osmprotection in plants [44,45]. The GABA-induced accumulations of WSC and proline were positively related to the enhanced heat tolerance of creeping bentgrass under controlled conditions [46]. Exogenous CTS induced the accumulation of soluble sugars for improved OA in white clover under drought stress and creeping bentgrass under salt stress [20,22]. In response to summer stress in 2017, 2018, and 2019, the CTS-, GABA-, and NaCl-treated turfs maintained a significantly higher WSC content and proline content, as well as a lower OP, than untreated turfs. These findings indicated that the enhanced thermotolerance of creeping bentgrass turfs regulated by exogenous GABA, CTS, and NaCl was related to better OA during summer months. However, an earlier study found that the exogenous application of NaCl ameliorated water transport and OA in white clover, mainly by improving WUE and the accumulation of aquaporins while reducing Tr and accumulations of WSC and proline. The possible explanation is that NaCl pretreatment provides many available Na^+^ ions for OA instead of going to the synthesis of WSC and proline for osmotic balance in cells under drought stress since the biosynthesis of organic metabolites is an energy-consuming process [29]. Differential effects of NaCl on regulating OA could vary greatly from one plant species to another under different abiotic stresses.

Heat stress causes cell membrane lipid peroxidation in the leaves of creeping bentgrass because of the excessive accumulation of reactive oxygen species, such as H_2_O_2_, as mainly manifested by the over-production of MDA [1,32]. The change in the EL has been widely used to evaluate cell membrane stability when creeping bentgrass undergoes heat stress [1]. Under controlled heat stress conditions, the foliar application of CTS activated multiple antioxidant enzymes in the leaves of creeping bentgrass, helping to mitigate heat-induced oxidative damage [23]. Foliar CTS pretreatment also significantly inhibited the leaf senescence of creeping bentgrass by minimizing the EL and accumulations of H_2_O_2_ and MDA [4]. The supply of GABA in the rhizosphere significantly improved the thermotolerance of creeping bentgrass associated with significant increases in antioxidant capacity and cell membrane stability in growth chambers [40]. During the summer months of 2017, 2018, and 2019, sustained high temperatures led to gradual increases in H_2_O_2_ content, MDA content, and the EL in the leaves of creeping bentgrass, which indicated that turf suffered from severe oxidative damage in summer. However, the foliar application of NaCl, GABA, or CTS significantly reduced the EL, H_2_O_2_ content, and MDA content in creeping bentgrass in summer, suggesting similar effects of three PGRs on mitigating oxidative damage and maintaining cell membrane stability. Earlier studies showed that white clover seeds primed with a low dose of NaCl effectively promoted germination related to enhanced antioxidant defense to reduce the EL level and accumulations of H_2_O_2_ and MDA under water-deficit conditions [28]. The exogenous application of low doses of NaCl also reduced drought-induced increases in the EL and MDA in white clover plants [29]. These previous studies, together with our current findings, suggest that a low dose of NaCl regulates the heat tolerance of creeping bentgrass and could be associated with reduced oxidative damage and improved cell membrane stability.

## 4. Materials and Methods

### 4.1. Plant Materials and Treatments

**Figure 9:**
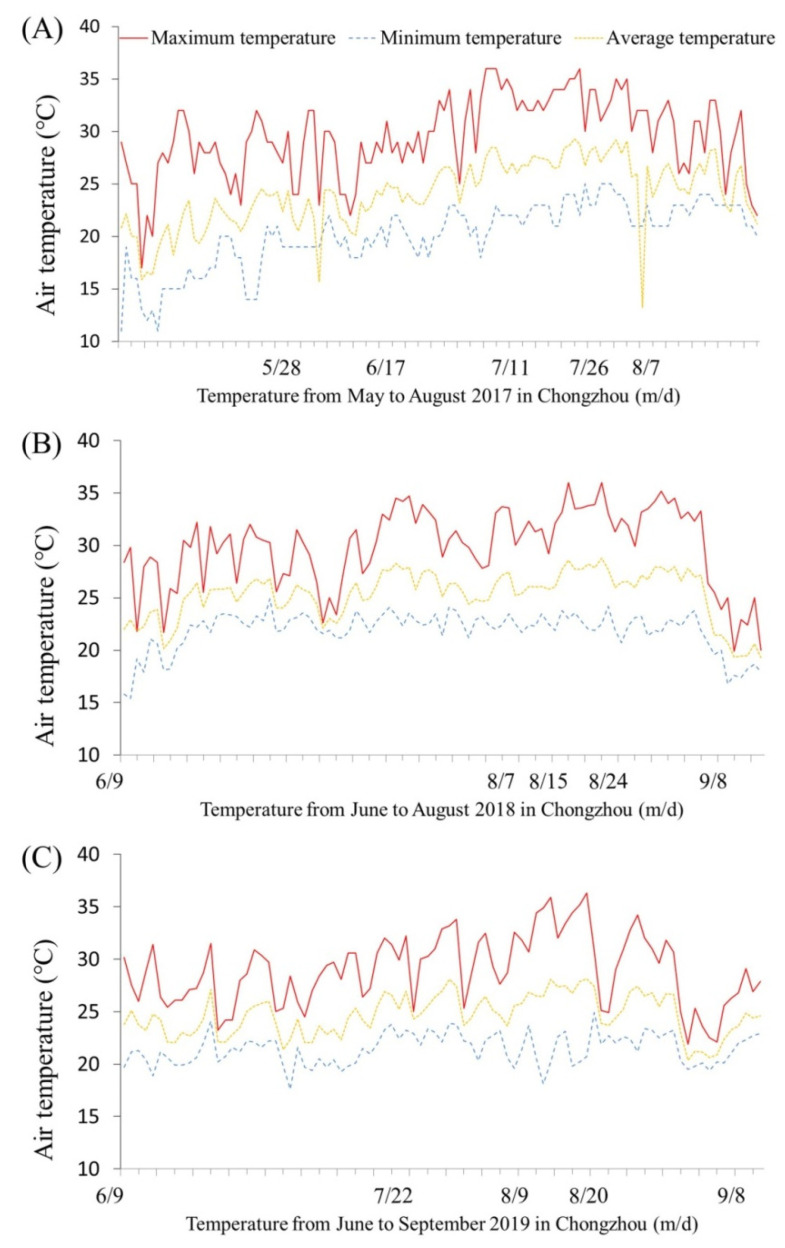
Daily maximum, minimum, and average air temperature in (**A**) May–August 2017, (**B**) June–September 2018, and (**C**) June–September 2019.

The experimental field is located in Sichuan Agricultural University Modern Research Base (30°56′ N, 103°65′ E). It is a typical subtropical monsoon climate with an annual average temperature of 16 °C, annual average sunshine hours of 1162 h, and an annual average rainfall of 1012 mm. Soil pH was 6.3, and soils included average organic matter 38 g·kg^−1^, alkaline nitrogen 136 mg·kg^−1^, total nitrogen 2 g·kg^−1^, available phosphorus 10 mg·kg^−1^, and available potassium 100 mg·kg^−1^ before planting turfgrass. Seeds of creeping bentgrass cv. Penncross (10 g·m^−2^) were sown evenly in test plots (2 m × 2 m) on 19 September 2016. The irrigation, fertilizer application, and daily turf management were consistent with our previous research [9]. After six months of establishment, the coverage reached 100%, and the mowing height of turfgrass was maintained at 1.0 cm until the foliar application of PGRs. Changes in the daily maximum, minimum, and average air temperature during the hot summers of 2017, 2018, and 2019 are shown in Figure 9A–C. The total number of days with maximum temperatures above 30 °C in the summers of 2017, 2018, and 2019 were 64, 59, and 45 days, respectively (Figure 9A–C). For the foliar application of PGRs, each test plot was sprayed with water as a non-PGR-treated control, 584 mg·L^−1^ NaCl solution (NaCl), 52 mg·L^−1^ GABA solution (GABA), or 100 mg·L^−1^ CTS solution (CTS). Each treatment was repeated 3 times (3 test plots), and a total of 12 test plots were arranged in the field based on a randomized complete block design. The optimal concentrations of NaCl, GABA, and CTS were the same as in our previous studies [15,20,29]. PGRs were applied on the turf in 2017 (23 May, 24 May, 25 May, 9 June, 23 June, 7 July, 21 July, and 4 August), 2018 (6 June, 7 June, 8 June, 22 June, 6 July, 20 July, 3 August, 17 August, and 31 August), and 2019 (6 June, 7 June, 8 June, 22 June, 6 July, 20 July, 3 August, 17 August, and 31 August). The spraying amount of water, NaCL, GABA, or CTS solution was 1 L for each test plot. Leaf samples were taken in 2017 (26 May, 17 June, 11 July, 26 July, and 7 August), 2018 (9 June, 7 August, 15 August, 24 August, and 8 September), and 2019 (9 June, 22 July, 9 August, 20 August, and 8 September), separately (Figure 9A–C). Table 2 shows the source, purity, and prices of GABA, CTS, and NaCl.

### 4.2. Measurements of Turf Quality, Chlorophyll Content, and Photosynthetic Characteristics

The color, coverage, and uniformity of turf quality (TQ) were evaluated according to the 9-point evaluation system of the National Turf Evaluation Program (NTEP). Nine points indicate the highest TQ, and one point represents the turf that is completely dead or dormant [47]. Chl was extracted in dimethyl sulfoxide until green disappeared, and the extract was measured at 663 nm and 645 nm [48]. Photosynthetic characteristics, including the net photosynthetic rate (Pn), transpiration rate (Tr), and water use efficiency (WUE) of leaves, were detected using the portable plant photosynthesis analyzer (CIRAS-3, PP Systems, Amesbury, MA, USA). Chl fluorescence parameters of photosystem II, including photochemical efficiency (Fv/Fm) and the performance index on an absorption basis (PI_ABS_), were detected using a CIRAS Pocket PEA fluorometer (Amesbury, MA, USA) [9].

### 4.3. Measurements of Water Status and Osmolyte Content

For relative water content (RWC) in leaves, 0.3 g of fresh leaves (FW) was collected from the turf and placed in 20 mL of deionized water for 12 h at 4 °C. After the leaves were saturated with deionized water, the saturated fresh weight (TW) was weighed. These leaves were then baked in an oven until reaching a constant weight. The dry weight (DW) was weighed. The RWC was calculated based on the formula RWC (%) = (FW − DW)/(TW − DW) × 100% [49]. For osmotic potential (OP) in leaves, 0.3 g of leaves were placed in a centrifuge tube filled with 20 mL of deionized water in the dark at 4 °C for 12 h. After wiping off moisture on the leaf surface, leaf saps were squeezed out. The solute concentration of leaf saps was measured as the c (mmol·kg^−1^) using a 4420 osmometer (Wescor, Inc., Logan, UT, USA), and the OP was calculated according to the formula OP (Mpa) = −c × 2.58 × 10^−3^ [50]. The proline content or water-soluble carbohydrate (WSC) content was determined according to the method by Bates [51] or the method in [52] with some modifications, respectively. Assay methods have been clearly demonstrated in our previous studies [1,46].

### 4.4. Measurement of Oxidative Damage and Cell Membrane Stability

To assess malonaldehyde (MDA) content in leaves, 0.2 g of fresh leaves were mixed with 1 mL of 50 mM cold phosphate buffer and then crushed and homogenized at 4 °C. After these mixtures were centrifuged at 10,000× *g* at 4 °C for 15 min, 1 mL of the reaction solution (20% *w*/*v* trichloroacetic acid and 0.5% *w*/*v* thiobarbituric acid) was mixed with 0.5 mL of the supernatant and then placed in boiling water for 20 min. The absorbance of the supernatant was measured at 532 nm and 600 nm [53]. For hydrogen peroxide (H_2_O_2_) content, 0.2 g of fresh leaves were homogenized in 1.5 mL of 0.1% TCA at 4 °C. The mixture was then centrifuged at 12,000× *g* at 4 °C for 15 min. A total of 0.5 mL of the supernatant was mixed with 0.5 mL of 10 mM potassium phosphate and 1 mL of 1 M potassium iodide, and then the mixture was placed in the dark for 10 min. The absorbance of the reaction mixture was read at 390 nm [54]. To detect electrolyte leakage (EL), fresh leaves (0.1 g) were soaked in 15 mL of ddH_2_O at room temperature for 24 h, and the initial conductivity (C_initial_) was measured using a conductivity meter (DDS-307A, Shanghai Precision Scientific Instrument Co., Ltd., Shanghai, China). These leaf samples were autoclaved at 120 °C for 20 min and cooled to room temperature to detect the maximum conductivity (C_max_). The EL was calculated as the percentage of C_initial_ in C_max_ [55].

### 4.5. Statistics and Data Analysis

SPSS 20 (SPSS Institute, IBM, Armonk, NY, USA, 2018) software was used to perform significant differences among four treatments based on multi-factor analysis of variance (ANOVA) together with the Tukey test at a probability level of 0.05. The subordinate function value analysis (SFVA) was used to comprehensively evaluate and sort the beneficial effects of CTS, GABA, and NaCl based on changes in TQ, Chl content, PI_ABS_, Fv/Fm, Pn, Tr, WUE, RWC, WSC content, proline content, EL, H_2_O_2_ content, MDA content, and OP during the hot summers of 2017, 2018, or 2019. A bigger SFVA indicated better tolerance to summer heat stress [56].

## 5. Conclusions

The triennial field tests in the subtropical zone found that the TQ of the creeping bentgrass significantly decreased during the hot summer months in 2017, 2018, and 2019. The foliar application of three exogenous PGRs (CTS, GABA, or NaCl) could significantly mitigate SBD in summer. The CTS, GABA, and NaCl exhibited similar positive effects on alleviating summer stress-induced heat damage to creeping bentgrass turfs by improving the accumulation of osmolytes, photochemical efficiency, Pn, Tr, and WUE and also reducing Chl loss, oxidative damage, and OP, contributing to osmotic homeostasis, photosynthetic performance, and the stability of the cell membrane system. Based on the comprehensive evaluation of the SFVA, the CTS had the best effect on the mitigation of SBD, followed by GABA and NaCl in 2017, 2018, and 2019. The current findings indicate that the foliar application of GABA, CTS, or NaCl provides a cost-effective strategy for mitigating SBD.

## Figures and Tables

**Table 1:** Comprehensive evaluation on the effect of CTS, GABA, or NaCl on alleviating SBD based on the subordinate function value analysis (SFVA).

Treatment	2017		2018		2019	
	SFV	Order	SFV	Order	SFV	Order
Water	0.32	4	0.27	4	0.25	4
NaCl	0.56	3	0.52	3	0.57	3
GABA	0.60	2	0.56	2	0.58	2
CTS	0.62	1	0.62	1	0.70	1

**Table 2:** Reagent price and cost.

Name	Source	Purity	Price	Cost
			(USD·g^−1^)	(USD·m^−2^·time^−1^)
GABA	Sigma-Aldrich Trading Co., Ltd. (Shanghai, China)	99%	1.9822	0.0256
CTS	Weifang Dongxing Chitosan Factory (Weifang, China)	90%	0.0445	0.0050
NaCl	Chengdu Kelong Chemical Co., Ltd. (Chengdu, China)	99%	0.0018	0.0003

## Data Availability

All of the datasets supporting the results of this article are included within the article.
